# Mental distress along the cascade of care in managing hypertension

**DOI:** 10.1038/s41598-022-20020-1

**Published:** 2022-09-23

**Authors:** Chiew Way Ang, Min Min Tan, Till Bärnighausen, Ulrich Reininghaus, Daniel Reidpath, Tin Tin Su

**Affiliations:** 1 South East Asia Community Observatory (SEACO) & Global Public Health, Jeffrey Cheah School of Medicine and Health Sciences, Monash University Malaysia, Subang Jaya, Selangor Malaysia; 2 Heidelberg Institute of Global Health, Faculty of Medicine, University of Heidelberg, Heidelberg, Baden-Württemberg Germany; 3 Department of Global Health and Population, Harvard T.H. Chan School of Public Health, Boston, MA USA; 4 Department of Public Mental Health, Central Institute of Mental Health, Medical Faculty Mannheim, University of Heidelberg, Mannheim, Germany; 5 ESRC Centre for Society and Mental Health, King’s College London, London, UK; 6 Centre for Epidemiology and Public Health, Health Service and Population Research Department, Institute of Psychiatry, Psychology and Neuroscience, King’s College London, London, UK; 7 Institute for Global Health and Development, Queen Margaret University, Edinburgh, EH21 6UU UK

**Keywords:** Psychology, Health care, Health services, Public health, Hypertension

## Abstract

Hypertension might be a contributing factor of mental illness. The aim of this study was to investigate the association between different levels of hypertension care and mental distress among hypertensive individuals in Malaysia. We constructed a hypertension care cascade using data of 6531 hypertensive individuals aged ≥ 35 years that were collected as part of the community health survey conducted in 2013 in the South East Asia Community Observatory. We examined the association between the status of hypertension care and mental distress using multiple logistic regressions. Respondents who had not been screened for hypertension and those who had uncontrolled blood pressure (BP) had higher odds of depression, anxiety and, stress compared to those who had been screened and those who had controlled BP, respectively. Respondents who were not taking antihypertensive medication had lower odds of depression and anxiety compared to those who were on medication. There was an association between different levels of hypertension care and mental distress. The application of a hypertension care cascade may help improve the provision of mental health support in primary care clinics. Specific mental health interventions could be provided for patients with particular needs along the cascade.

## Introduction

Depression affects 264 million people of all ages globally^1^, and depressive symptoms are one of the top 20 leading causes of reduced disability-adjusted life years (DALYs)^2^. Around 27% and 23% of the population in Southeast Asia are living with depression and anxiety, respectively^3^. In Malaysia, depression and anxiety disorders are among the top ten causes of disease burden that have contributed to DALYs since 2010^4^. Among Malaysians aged 16 and above, 1.7% experienced generalized anxiety disorder (GAD)^4,5^. The prevalence of Malaysian adults experiencing depression had increased from 1.8% in 2015 to 2.3% in 2019^6^.

Mental health issues result from a complex interaction of social, psychological and, biological factors^1^. Physical illness such as hypertension might be one of the contributing factors to mental illness^1^. Several studies have demonstrated associations between mental health and hypertension. Anxiety disorder^7,8^ and depression^7,9^ were linked with hypertension, while a systematic review concluded that mental illness was associated with increased blood pressure variability in younger and middle-aged adults^10^. However, other studies found no association between the diagnosis of hypertension and depression^11,12^. Hence, it is important to investigate the association of mental health across the hypertension care continuum to reduce mortality rate and improve quality of life.

In Malaysia, three in ten adults aged 18 and above, or 6.4 million, have hypertension^6^. Although the awareness of and controls for hypertension have improved over time, the prevalence of awareness, treatment, and, control for hypertension remains low in the country^6,13^. About half of the hypertensive individuals in Malaysia were aware that they have hypertension, and among those who were aware, 90% of them were taking anti-hypertensive medication. However, only 45% had their blood pressure (BP) controlled. While many studies in Malaysia examined hypertension risk factors and the psychological determi^6^nants of hypertension among adolescents, adults and, elderly^14–18^, studies that investigate mental health well-being along the cascade of hypertension care are lacking.

Hypertension care cascade has been used to depict where along the care process (screening, diagnosis, treatment and control status) patients are lost^19^, inform policymakers, assess health system performance, and improve the efficacy of health intervention^20,21^. Besides providing a baseline in hypertension surveillance, the hypertension care cascade plays an important role as the supporting information in developing an effective intervention for improving hypertension control^22,23^. Monitoring the pattern of hypertensive patients across the care continuum is important to identify the proper strategy to improve hypertension control for the country^22^. While past studies have shown that various socio-demographic factors are associated with the different levels of hypertension care^19–21^, none have examined the intersection of mental health and the hypertension care continuum. In this study, we aimed to (1) determine the cascade of care for hypertension in Malaysia and (2) investigate the association between different levels of hypertension care and mental health among hypertensive individuals. Further details of the different levels of hypertension care will be explained in the methodology section.

## Methods

### Study design

This was a cross-sectional study utilizing secondary data collected as part of the community health survey in 2013 in the Southeast Asia Community Observatory (SEACO), a health and demographic surveillance system (HDSS) established by Monash University in Segamat district Johor state, Malaysia^24^. All individuals aged five and above living in sub-districts where SEACO operates were invited to the survey and interviewed by trained data collectors. The survey included questionnaire items about demographic and socio-economic characteristics (e.g. education, age, ethnicity, and income), self-reported health conditions (e.g. diabetes status, hypertension status), and mental health status.

### Participants and data collection

The total number of respondents in the survey was 25,184^24^. Health screening (anthropometric measurements, blood pressure and random glucose) was only conducted among respondents aged 35 years and above (N = 13,831) to measure height, weight, blood pressure, and random blood glucose. Only those aged 35 and above and who reported having hypertension (see “Definition of hypertension” in measures) were included (N = 6531).

All procedures involving human subjects were approved by the Monash University Human Research Ethics Committee (MUHREC) (Project ID: 13142). All methods were performed following the relevant guidelines and regulations. All participants had signed and provided an informed consent form.

### Measures

#### Mental health

Mental health was measured by the Depression, Anxiety, Stress Scale-21 (DASS-21), a self-reported inventory to measure depression, anxiety, and stress^25–27^. DASS-21 is a shorter version of the 42-item DASS (DASS-42). Respondents were asked to rate the extent to which they experienced depression, anxiety and stress over the past week. The depression subscale assessed hopelessness and devaluation of life; the anxiety subscale measured skeletal muscle effect and autonomic arousal^25,26,28^, and the stress subscale focused on chronic non-specific arousal^25,26,28^. Individual scale items were scored on a 4-point response scale (never, sometimes, often, almost always) of frequency or severity of respondents’ experiences. The score of each subscale was summed and multiplied by two so that it was comparable to DASS-42, and a higher score indicated more severe symptoms. The categories for severity suggested by Lovibond and Lovibond^26^ included normal, mild, moderate, severe and extremely severe. However, due to the small number of individuals with a highly severe state in our study, we re-coded DASS into two categories: normal, and at least mild.

DASS-21 has been widely used in both clinical and non-clinical contexts^27,29,30^. We used the validated Malay version of the DASS-21 since Malay is the national language of Malaysia. The subscales have acceptable internal consistency (Cronbach’s α = 0.84, 0.74, and 0.79 for the depression, anxiety and stress subscales, respectively)^31^. In this study, the internal consistency of DASS-21 was 0.92, 0.87, and 0.89 for the depression, anxiety and stress subscales, respectively.

#### Definition of hypertension and construction of hypertension care cascade

Systolic blood pressure (SBP) and diastolic blood pressure (DBP) were measured using the Omron HEM 7120 E Blood Pressure Monitor M2 Basic Digital Intellisense. The respondents were seated and rested for 15 min before their BP was measured by trained data collectors^32^. Three readings of BP were taken at a five-minute interval, and the average of the BP readings was calculated.

The respondents answered questionnaire items adapted from the WHO STEPwise manual, which included “Have you ever had your blood pressure measured by a doctor or other health worker?”, “Have you ever been told by a doctor or other health worker that you have raised blood pressure?” and “Have you taken any drugs (medication—not Traditional Chinese Medicine (TCM) in the past 2 weeks?”^32^. Hypertension was defined as SBP ≥ 140 mm/Hg or DBP ≥ 90 mmHg or responding with “yes” to the question “Have you ever been told by a doctor or other health worker that you have raised blood pressure?”.

We calculated the proportions of (1) respondents who had their BP measured by a doctor or other healthcare worker (screened, N = 4725), and those who had not (unscreened, N = 1806); (2) respondents who reported having been told by doctor or other healthcare worker that they had raised BP or hypertension (diagnosed, N = 3259), and those who had not been told (undiagnosed, N = 3272); (3) respondents who were taking anti-hypertensive medication (treated, N = 2510), and those who were not taking anti-hypertensive medication (not in treatment, N = 4021); (4) respondents who had been diagnosed with hypertension and were taking anti-hypertensive medication (diagnosed and treated, N = 2510), and those diagnosed with hypertension and but were not taking any anti-hypertensive medication (diagnosed and untreated, N = 749); and (5) respondents who were taking anti-hypertensive medication and whose DBP was below 90 mm/Hg and SBP was below 140 mm/Hg (treated and controlled, N = 966), and those were taking anti-hypertensive medication but whose DBP exceeded 90 mm/Hg and/or SBP exceeded 140 mm/Hg (treated and uncontrolled, N = 1544); if the patients reported having diabetes or kidney diseases, then the cut-off point for controlled DBP and SBP was < 79 mm/Hg and/or < 129 mm/Hg, respectively.

#### Socio-demographic, diabetes status and Body Mass Index (BMI)

Covariates were selected a priori based on their availability and likelihood to confound associations between hypertension status and mental health conditions. Previous studies have demonstrated the potential confounding role of sociodemographic^17,33–35^ as well as cardiometabolic risk factors of BMI^36–39^ and diabetes^17,34,40^ within the hypertension and mental health relationship. Socio-demographic factors included age (35–49, 50–59, 60–69, 70 and above), gender (male, female), ethnicity (Malay, Chinese, Indian, Aborigine, Others), marital status (never married, married, separated/divorced, widowed/widower, others [e.g. separated, widowed]), income (below RM 1000, RM 1000–RM 1999, RM 2000–RM 2999, RM 3000 and above), education (no formal education, primary, secondary, tertiary, others [e.g. religious school, no formal education]). Self-reported diabetes status (yes, no) was ascertained from the question “Have you been told by a doctor or a health worker that you have raised blood sugar or diabetes?” while BMI was calculated as weight divided by height squared (kg/m^2^) and categorised as underweight/normal (< 25), overweight (25–29.9) and obese (30 and above).

### Statistical analysis

Descriptive statistics were presented using proportions for categorical variables. Chi-square tests were conducted to determine the bivariate associations between mental health indicators (depression, anxiety, and stress) and selected independent variables. Binary logistic regression was conducted to determine association between hypertension care and mental health, unadjusted and adjusted for age, sex, ethnicity, marital status, education, income, BMI and diabetes status. Multicollinearity was checked by using the collinearity matrix and variance inflation factor (VIF). As the dataset in this study was a secondary data that obtained from a well-established HDSS platform which had recruited a large number of respondents (more than 6500) for this analysis, thus, the sample size was sufficient and appropriate to conduct logistic regression. We examined depression, anxiety, and stress in separate models (15 models; 5 hypertension care continuum with 3 mental health indicator each). Adjusted odds ratios were presented to determine the effect of hypertension care cascade on mental health, controlling for socio-demographic, diabetes status and Body Mass Index (BMI). Data were analysed using SPSS version 20.

## Results

A total of 6531 hypertensive individuals were included in this study. The mean age of the respondents was 58.8 years (SD = 11.6). Overall, about 78% of respondents were aged 50 and above (pre-elderly and elderly cohort/older age cohort) and around 55% were female. Majority respondents that were interviewed for this study was aged between 50 and 59 years old (consists of 28.1% males and 33.7% females) and 60–69 years old (consists of 32.0% males and 25.1% females). The majority of the respondents were Malay (63.4%), followed by Chinese (25.3%) and Indians (9.2%). Most respondents were married (78.9%) and had primary or secondary education (88%). Seventy-eight percent reported monthly household income that was less than RM 2000 (USD666.70) while 39% were overweight. The percentage of respondents with at least mild depression, anxiety and stress were 16.0%, 21.2% and 7.3%, respectively. Respondents’ characteristics are displayed in Table 1.

**Table 1:** Socio-demographics, health-related characteristics, and mental health status of respondents (N = 6531).

Age mean (SD)				58.81 (11.61)
Variables				
Age group	Gender			
	Male		Female	
	n	%	n	%
35–49	632	21.7	795	22.0
50–59	819	28.1	1218	33.7
60–69	932	32.0	909	25.1
70 and above	532	18.3	694	19.2

Variables	n		%	
**Age group**				
35–49	1427		21.8	
50–59	2037		31.2	
60–69	1841		28.2	
70 and above	1226		18.8	
**Gender**				
Male	2915		44.6	
Female	3616		55.4	
**Ethnicity**				
Malay	4142		63.4	
Chinese	1655		25.3	
Indian	604		9.2	
Aborigine	99		1.5	
Other	31		0.5	
**Marital status**				
Never married	266		4.1	
Married	5147		78.9	
Others	1107		16.9	
**Education**				
Primary	3139		49.2	
Secondary	2477		38.8	
Tertiary	197		3.0	
Others	571		8.7	
**Monthly household income**^†^				
Below RM 1000	2416		45.9	
RM 1000–RM 1999	1689		32.1	
RM 2000–RM 2999	607		11.5	
RM 3000 and above	551		10.5	
**Known diabetes status**				
No	5301		81.3	
Yes	1223		18.7	
**BMI**				
Underweight/Normal	2124		34.3	
Overweight	2396		38.7	
Obese	1672		27.0	
**Depression**				
Normal	5397		84.0	
At least mild	1029		16.0	
**Anxiety**				
Normal	5098		78.8	
At least mild	1371		21.2	
**Stress**				
Normal	5982		92.7	
At least mild	472		7.3	

BMI, Body Mass Index.

Proportion are percentage of non-missing data.

^†^USD1 is equivalent to RM 3.00 (13th May 2013).

**Figure 1:**
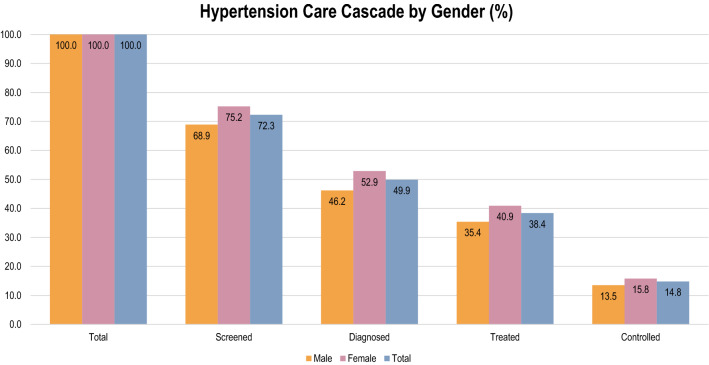
Hypertension care cascade by sex. Distribution of total, screened, diagnosed, treated and controlled hypertension care continuum by gender in percentage (denominator: hypertension population by each gender).

The hypertension care cascade is presented in Fig. 1 and Fig. S1 and summarized in Table S2. Figure S1 displays the hypertension care cascade by gender, using the previous care continuum as denominator while Fig. 1 presents the hypertension care cascade with the hypertension population in the dataset as denominator. Among the hypertensive respondents, 72.3% had been screened for hypertension, a 27.7% loss. Of those who have ever had their blood pressure measured, 69.0% were diagnosed with hypertension, a 31.0% loss. Meanwhile, among those who were diagnosed, 77.0% had taken blood pressure medication, a 23.0% loss. Of those who had taken blood pressure medication, only 38.5% had controlled blood pressure, a 61.5% loss (refer to Fig. S1). Overall, female respondents had higher prevalence of hypertension. While significantly more females were screened, diagnosed and treated for hypertension, there was no significant difference between males and females in blood pressure control (*p*-value = 0.791) (refer to Table S2). About 72% of respondents (69% males and 75% females) reported that they had their BP measured by a doctor or healthcare worker. Among the female respondents, approximately 53% (vs 46% male) were aware that they had hypertension, while 41% (vs 35% male) reported taking anti-hypertensive medication. However, only about 16% (vs 13.5% male) had their BP under control.

Tables 2, 3 and 4 summarises the socio-demographic, diabetes status, and BMI categories of the respondents by mental health indicators (depression, anxiety and stress). Depression, anxiety and stress were more likely reported by the younger age (35–49), male, never married, secondary education, higher income (RM 2000 and above), not diabetic and lower BMI groups. For ethnicity however, Chinese reported the highest proportion for stress while Malay reported higher proportion in anxiety. Tables 5, 6 and 7 summarises the prevalence of depression, anxiety and stress across the hypertension care continuum. The prevalence of having depression, anxiety and stress was significantly higher among respondents who never had their BP measured by a doctor or a healthcare worker (not screened), who were unaware of their hypertension (undiagnosed), and who did not have their hypertension treated (untreated, including those aware and unaware of their hypertension). Respondents who underwent treatment reported a significantly higher prevalence of depression, anxiety, and stress among those who were diagnosed with hypertension. Those with uncontrolled BP had a higher prevalence of depression, anxiety and stress than those with their BP controlled.

**Table 2:** Socio-demographics and diabetes status by depression.

	Depression^a^		
	Normal (n = 5397)	At least mild (n = 1029)	${\upchi }^{2}$, *p*-value
	n (%)	n (%)	
**Age group**			
35–49	1121 (79.7)	286 (20.3)	24.930, *p* < 0.001
50–59	1706 (85.2)	296 (14.8)	
60–69	1544 (85.2)	268 (14.8)	
70 and above	1026 (85.1)	179 (14.9)	
**Gender**			
Male	2368 (82.7)	497 (17.3)	6.843, *p* = 0.009
Female	3029 (85.1)	532 (14.9)	
**Ethnicity**			
Malay	3428 (84.1)	650 (15.9)	0.334, *p* = 0.988
Chinese	1355 (83.6)	266 (16.4)	
Indian	506 (84.5)	93 (15.5)	
Aborigine	82 (84.5)	15 (15.5)	
Other	26 (83.9)	5 (16.1)	
**Marital status**			
Never married	183 (70.4)	77 (29.6)	49.085, *p* < 0.001
Married	4246 (83.8)	818 (16.2)	
Others	962 (88.0)	131 (12.0)	
**Education**			
Primary	2623 (84.7)	473 (15.3)	41.391, *p* < 0.001
Secondary	1995 (81.9)	440 (18.1)	
Tertiary	167 (85.2)	29 (14.8)	
Others	516 (92.8)	40 (7.2)	
**Monthly household income**^**†**^			
Below RM 1000	2150 (90.0)	239 (10.0)	154.029, *p* < 0.001
RM 1000–RM 1999	1418 (85.3)	245 (14.7)	
RM 2000–RM 2999	457 (75.8)	146 (24.2)	
RM 3000 and above	395 (72.5)	150 (27.5)	
**Known diabetes status**			
No	4295 (82.3)	922 (17.7)	57.084, *p* < 0.001
Yes	1097 (91.2)	106 (8.8)	
**BMI**			
Underweight/Normal	1612 (77.3)	473 (22.7)	112.531, *p* < 0.001
Overweight	2010 (85.2)	350 (14.8)	
Obese	1483 (89.9)	167 (10.1)	

BMI, Body Mass Index.

Values in parentheses are the percentage of the cases.

^†^USD1 is equivalent to RM 3.00 (13th May 2013).

^a^The total number of respondents answered the assessment of depression was 6426.

**Table 3:** Socio-demographics and diabetes status by anxiety.

	Anxiety^a^		
	Normal (n = 5098)	At least mild (n = 1371)	${\upchi }^{2}$, *p*-value
	n (%)	n (%)	
**Age group**			
35–49	1084 (76.7)	329 (23.3)	9.351, *p* = 0.025
50–59	1586 (78.6)	432 (21.4)	
60–69	1440 (78.8)	387 (21.2)	
70 and above	988 (81.6)	223 (18.4)	
**Gender**			
Male	2250 (77.8)	642 (22.2)	3.168, *p* = 0.075
Female	2848 (79.6)	729 (20.4)	
**Ethnicity**			
Malay	3149 (76.7)	957 (23.3)	31.833, *p* < 0.001
Chinese	1338 (81.9)	295 (18.1)	
Indian	500 (83.3)	100 (16.7)	
Aborigine	86 (86.9)	13 (13.1)	
Other	25 (80.6)	6 (19.4)	
**Marital status**			
Never married	180 (68.7)	82 (31.3)	29.639, *p* < 0.001
Married	4002 (78.4)	1100 (21.6)	
Others	912 (83.3)	183 (16.7)	
**Education**			
Primary	2458 (78.8)	662 (21.2)	20.575, *p* < 0.001
Secondary	1904 (77.7)	546 (22.3)	
Tertiary	156 (80.0)	39 (20.0)	
Others	484 (86.3)	77 (13.7)	
**Monthly household income**^**†**^			
Below RM 1000	2063 (85.9)	339 (14.1)	112.794, *p* < 0.001
RM 1000–RM 1999	1304 (77.7)	374 (22.3)	
RM 2000–RM 2999	436 (72.4)	166 (27.6)	
RM 3000 and above	385 (70.1)	164 (29.9)	
**Known diabetes status**			
No	4036 (76.9)	1215 (23.1)	64.190, *p* < 0.001
Yes	1058 (87.3)	154 (12.7)	
**BMI**			
Underweight/Normal	1509 (72.0)	586 (28.0)	97.199, *p* < 0.001
Overweight	1900 (80.0)	476 (20.0)	
Obese	1413 (85.0)	249 (15.0)	

BMI, Body Mass Index.

Values in parentheses are the percentage of the cases.

^†^USD1 is equivalent to RM 3.00 (13th May 2013).

^a^The total number of respondents answered the assessment of anxiety was 6469.

**Table 4:** Socio-demographics and diabetes status by stress.

	Stress^a^		
	Normal (n = 5982)	At least mild (n = 472)	${\upchi }^{2}$, *p*-value
	n (%)	n (%)	
**Age group**			
35–49	1253 (88.6)	161 (11.4)	45.719, *p* < 0.001
50–59	1881 (93.6)	129 (6.4)	
60–69	1719 (94.4)	102 (5.6)	
70 and above	1129 (93.4)	80 (6.6)	
**Gender**			
Male	2657 (91.9)	233 (8.1)	4.331, *p* = 0.037
Female	3325 (93.3)	239 (6.7)	
**Ethnicity**			
Malay	3829 (93.5)	266 (6.5)	13.029, *p* = 0.011
Chinese	1479 (90.8)	150 (9.2)	
Indian	555 (92.3)	46 (7.7)	
Aborigine	91 (92.9)	7 (7.1)	
Other	28 (90.3)	3 (9.7)	
**Marital status**			
Never married	207 (79.0)	55 (21.0)	99.184, *p* < 0.001
Married	4710 (92.6)	377 (7.4)	
Others	1059 (967)	36 (3.3)	
**Education**			
Primary	2964 (95.3)	145 (4.7)	97.196, *p* < 0.001
Secondary	2172 (88.8)	274 (11.2)	
Tertiary	181 (92.3)	15 (7.7)	
Others	537 (96.1)	22 (3.9)	
**Monthly household income**^**†**^			
Below RM 1000	2313 (96.7)	78 (3.3)	261.433, *p* < 0.001
RM 1000–RM 1999	1601 (95.5)	75 (4.5)	
RM 2000–RM 2999	516 (85.3)	89 (14.7)	
RM 3000 and above	443 (80.7)	106 (19.3)	
**Known diabetes status**			
No	4796 (91.6)	439 (8.4)	48.051, *p* < 0.001
Yes	1181 (97.4)	32 (2.6)	
**BMI**			
Underweight/Normal	1800 (86.0)	294 (14.0)	200.346, *p* < 0.001
Overweight	2257 (95.2)	114(4.8)	
Obese	1603 (96.9)	51 (3.1)	

BMI, Body Mass Index.

Values in parentheses are the percentage of the cases.

^†^USD1 is equivalent to RM 3.00 (13th May 2013).

^a^The total number of respondents answered the assessment of stress was 6454.

**Table 5:** Depression status across hypertension care continuum.

	Depression		
	Normal (n = 5397)	At least mild (n = 1029)	${\upchi }^{2}$, *p*-value
	n (%)	n (%)	
**Screened hypertension**			
Screened	4118 (88.6)	529 (11.4)	267.484, *p* < 0.001
Not screened	1279 (71.9)	500 (28.1)	
**Diagnosed hypertension**			
Diagnosed	2812 (87.8)	391 (12.2)	68.776, *p* < 0.001
Undiagnosed	2585 (80.2)	638 (19.8)	
**Hypertension treatment**			
Treated	2143 (87.0)	320 (13.0)	27.098, *p* < 0.001
Untreated	3254 (82.1)	709 (17.9)	
**Treatment status in diagnosed group**			
Treated	2143 (85.4)	320 (13.0)	6.130, *p* = 0.013
Untreated	669 (90.4)	71 (9.6)	
**BP status in treated group**			
Controlled	855 (90.5)	90 (9.5)	16.318, *p* < 0.001
Uncontrolled	1288 (84.8)	230 (15.2)	

BP, blood pressure.

Values in parentheses are the percentage of the cases.

**Table 6:** Anxiety status across hypertension care continuum.

	Anxiety		
	Normal (n = 5098)	At least mild (n = 1371)	${\upchi }^{2}$, *p*-value
	n (%)	n (%)	
**Screened hypertension**			
Screened	3911 (83.5)	774 (16.5)	222.076, *p* < 0.001
Not screened	1187 (66.5)	597 (33.5)	
**Diagnosed hypertension**			
Diagnosed	2656 (82.2)	575 (17.8)	44.600, *p* < 0.001
Undiagnosed	2442 (75.4)	796 (24.6)	
**Hypertension treatment**			
Treated	2006 (80.8)	477 (19.2)	9.485, *p* = 0.002
Untreated	3092 (77.6)	894 (22.4)	
**Treatment status in diagnosed group**			
Treated	2006 (80.8)	477 (19.2)	14.664, *p* < 0.001
Untreated	650 (86.9)	98 (13.1)	
**BP status in treated group**			
Controlled	806 (84.7)	146 (15.3)	14.934, *p* < 0.001
Uncontrolled	1200 (78.4)	331 (21.6)	

BP, blood pressure.

Values in parentheses are the percentage of the cases.

**Table 7:** Stress status across hypertension care continuum.

	Stress		
	Normal (n = 5982)	At least mild (n = 472)	${\upchi }^{2}$, *p*-value
	n (%)	n (%)	
**Screened hypertension**			
Screened	4448 (95.3)	219 (4.7)	170.793, *p* < 0.001
Not screened	1534 (85.8)	253 (14.2)	
**Diagnosed hypertension**			
Diagnosed	3050 (94.8)	168 (5.2)	41.464, *p* < 0.001
Undiagnosed	2932 (90.6)	304 (9.4)	
**Hypertension treatment**			
Treated	2329 (94.2)	143 (5.8)	13.810, *p* < 0.001
Untreated	3653 (91.7)	329 (8.3)	
**Treatment status in diagnosed group**			
Treated	2329 (94.2)	143 (5.8)	6.859, *p* = 0.009
Untreated	721 (96.6)	25 (3.4)	
**BP status in treated group**			
Controlled	903 (95.4)	44 (4.6)	3.651, *p* = 0.056
Uncontrolled	1426 (93.5)	99 (6.5)	

BP, blood pressure.

Values in parentheses are the percentage of the cases.

Multicollinearity test showed no correlation between the selected variables (VIF values ranged from 1.00 to 4.70 in all 15 models). Table 8 shows the association between hypertension care status and depression, anxiety and stress, adjusted for age, sex, ethnicity, marital status, education, income, diabetes status, and BMI. Respondents who did not have their BP measured by a doctor or a healthcare worker (not screened) had higher odds of experiencing depression (AOR = 2.027, 95% CI 1.692, 2.428), anxiety (AOR = 1.787, 95% CI 1.521, 2.099) and stress (AOR = 1.653, 95% CI 1.274, 2.144), compared to those who had their BP measured previously. Those who had not taken antihypertensive medication (regardless of diagnosis status) had lower odds of having anxiety (AOR = 0.793, 95% CI 0.675, 0.933) and stress (AOR = 0.740, 95% CI 0.565, 0.969). Among those diagnosed with hypertension, those who were not taking antihypertensive medication had lower odds of depression (AOR = 0.626, 95% CI 0.441, 0.888), anxiety (AOR = 0.542, 95% CI 0.402, 0.732) and stress (AOR = 0.580, 95% CI 0.339 m 0.991). Among respondents who were taking antihypertensive medication, those with uncontrolled BP had higher odds of depression (AOR = 2.118, 95% CI 1.543, 2.908), anxiety (AOR = 1.932, 95% CI 1.479, 2.522) and stress (AOR = 1.646, 95% CI 1.066, 2.540). Detailed results of each adjusted model along with the observed cases, $\chi^{2}$, − 2 log likelihood, R^2^, and goodness-of-fit are presented in Supplementary Tables S3–S7. The results above were similar to those from the unadjusted models for screened hypertension, the treatment status in diagnosed group, and BP status in the treated group (Supplementary Table S8).

**Table 8:** The association between hypertension care status and depression, anxiety and stress (Multivariable logistic regressions).

	Depression		Anxiety		Stress	
	AOR (95% CI)^a^	*p*-value	AOR (95% CI)^a^	*p*-value	AOR (95% CI)^a^	*p*-value
**Screened hypertension**						
Screened	REF		REF		REF	
Not screened	2.027 (1.692, 2.428)	*p* < 0.001	1.787 (1.521, 2.099)	*p* < 0.001	1.653 (1.274, 2.144)	*p* < 0.001
**Diagnosed hypertension**						
Diagnosed	REF		REF		REF	
Undiagnosed	1.144 (0.954, 1.372)	*p* = 0.146	0.975 (0.831, 1.143)	*p* = 0.751	0.875 (0.672, 1.138)	*p* = 0.319
**Hypertension treatment**						
Treated	REF		REF		REF	
Untreated	0.956 (0.795, 1.151)	*p* = 0.637	0.793 (0.675, 0.933)	*p* = 0.005	0.740 (0.565, 0.969)	*p* = 0.029
**Treatment status in diagnosed group**						
Treated	REF		REF		REF	
Untreated	0.626 (0.441, 0.888)	*p* = 0.009	0.542 (0.402, 0.732)	*p* < 0.001	0.580 (0.339, 0.991)	*p* = 0.046
**BP status in treated group**						
Controlled	REF		REF		REF	
Uncontrolled	2.118 (1.543, 2.908)	*p* < 0.001	1.932 (1.479, 2.522)	*p* < 0.001	1.646 (1.066, 2.540)	*p* = 0.024

The adjusted odd ratios of depression, anxiety and stress (outcome variables) in binary logistic regression analysis with five hypertension care status each (independent variables). The result of AOR in this table were obtained from 15 models in total (5 different hypertension care cascade with 3 mental health indicators each), please find the results of binary logistic regression model for all included variables in Supplementary Tables S3–S7 (for different hypertension care status with three mental health indicators) with the observed cases, ${\upchi }^{2}$, − 2 log likelihood, R^2^, and goodness-of-fit.

AOR, adjusted odd ratios; BMI, Body Mass Index; CI, confidence interval; REF, reference.

Values in parentheses are 95% of confidence interval.

^a^Adjusted for age, sex, ethnicity, marital status, education, income, diabetes status and BMI.

## Discussion

The current study shows an association between hypertension care and mental distress along the care cascade among hypertensive individuals. Specifically, those who had not been screened for hypertension were more likely to have depression, anxiety and stress. Our study is inconsistent with another study which found no association between unscreened hypertension and mental illness^41^. Screening for mental distress in conjunction with hypertension in the population might help to uncover those at risk for mental distress in this study.

Interestingly, individuals who were not under treatment for hypertension were less likely to report three forms of mental illness. These individuals might be unaware of the consequences of untreated hypertension and might be more relaxed about their untreated hypertension^11^. A study was done in China also found that hypertensive patients with mental disorders were more likely to receive treatment compared to those that did not have mental disorder^42^. Among those undergoing treatment for hypertension, those with uncontrolled BP had a higher risk of experiencing mental illness than those with their BP controlled. The result was consistent with past studies where patients with uncontrolled BP had a higher risk of developing depression^43–45^. The association between uncontrolled BP and a higher risk of developing mental illness may be due to clinical inertia, a lack of medication intensification, no specialist referral, or work-up for identifiable hypertension despite uncontrolled BP^46^. Hypertensive patients with mental illness might be less likely to seek medical treatment, resulting in uncontrolled BP. This showed the importance of medical adherence in controlling blood pressure among hypertensive patients with mental distress. Hypertensive patients may experience profound emotion which may be detrimental to the brain, increasing symptoms of mental distress^25,47^. Such a situation results in patient reluctance or treatment non-compliance that can lead to uncontrolled hypertension.

Moreover, a sense of hopelessness associated with poorer health status might increase the risk of developing mental illness among patients^48^. Uncontrolled BP might also be explained by medical non-adherence. Patients with lower medication adherence were more likely to be stressed^25^ and depressed^44,49^. Another study reported that hypertensive patients undergoing treatment might experience negative emotions or have pathological disorders in the brain, which increase the risk of mental distress symptoms^47^. These symptoms may result in the patient's inability to comply with the treatment (medical non-adherence)^47^. Therefore, detection and managing negative emotions/mental distress among hypertensive patients would lead to improved medication adherence, depressive symptoms and BP control^25,50^. Our findings also showed that while it is important to screen people for hypertension and encourage them to begin treatment if they are diagnosed, following up on hypertensive patients to ensure medical adherence is crucial in blood pressure control and improving mental well-being. Some studies^47,51^ found that there exist bidirectional association between hypertension and mental disorders whereby it was not well captured in this study due to the study design. Therefore, further analysis in examining the bidirectional association between hypertension and mental disorders is required.

The hypertensive individuals in our study have better hypertension control than other countries. The prevalence of hypertensive individuals screened for hypertension was 51% in South Africa^21^ and 69% in India^19^, respectively, which were lower than that in our study (72%). The prevalence of hypertensive individuals diagnosed with hypertension (50%) and treated (38%) for hypertension and who had their hypertension under control (15%) (refer to Fig. 1) in our study were also higher compared to the two countries (South Africa: 28% diagnosed, 22% treated, 9% control; India: 35% diagnosed, 10% treated, 5% control)^19,21^. The prevalence of hypertension care cascade in our study was also higher than in the past study among hypertensive patients in low-middle income countries (LMIC) (73.6% measured, 39.2% diagnosed, 29.9% treated and 10.3% control)^23^. The percentage of hypertensive individuals in our study who were aware of their hypertension (diagnosed hypertension) was similar to the findings from the National Health and Morbidity Survey (NHMS) 2019 (50%), a national health survey commissioned since 1986 by the Ministry of Health Malaysia to examine the health status and determinants of health of Malaysians^6^. The higher prevalence of diagnosed hypertension in our study compared to that reported in NHMS might be due to different study designs. NHMS is cross-sectional, while SEACO HDSS is longitudinal and regularly follows up on respondents through home visitations. Besides that, the percentage of diagnosed hypertension obtained in NHMS 2019 was among adults aged 18 and above while the percentage of diagnosed hypertension of this study was among adult aged 35 and above. However, similar to other studies, the percentage of hypertensive individuals screened, diagnosed and treated for hypertension and who had their hypertension under control decreased across each cascade. The significant loss to care across the hypertension cascade indicated that public awareness of hypertension and access to health care remained low. Thus, there is an urgent need to raise awareness about the importance of hypertension screening, medical adherence, and maintenance of healthy BP.

Compared with older respondents, the younger respondents (aged 35–49) in our study had a higher prevalence of mental distress, which might be explained by higher stress resulting from household responsibilities among the younger cohorts^33,52^. Chinese had the highest depression and stress prevalence while Malays had the highest anxiety prevalence compared with other ethnic groups. This finding was contrary to previous studies conducted in Malaysia, where Indians were found to have the highest risk of mental illness^33–35^. Another study showed that Malays and Chinese had a lower risk of experiencing mental health disorders compared with Indians^53^. In another study, higher-income Chinese were more likely to report depression due to work pressure^52^. The prevalence of depression, anxiety and stress was higher among males than females. This is consistent with a past study and might be due to men being the main financial supporter in the family and thus experiencing more work-related stress^53^.

Moreover, respondents who had never married in our study had a higher prevalence of mental distress compared to those who were married. This might be due lack of social support from an intimate partner^52^. The practice of cohabitation is rarely seen in rural Asia. The current study also found that respondents with lower education attainment had a higher mental distress prevalence than those with tertiary education. Previous studies have shown that low educational attainment is one of the risk factors for mental illness^33,34,52^, which could be explained by inadequate health literacy and lack of knowledge of the cause of hypertension and preventive measures^34^. The prevalence of mental distress was higher among respondents without diabetes compared to those with diabetes, which contradicts previous studies where outpatients^53^ and diabetic patients^34^ with good self-rated health or healthy respondents had less likelihood of reporting mental health disorders. Respondents in underweight/normal BMI category were more likely to have all three mental health conditions, contrary to the other studies whereby overweight or obese individuals had higher tendency of experiencing mental illness^38,39,54^. However, a study in Australia found that people who were underweight had poorer mental health but the explanation of this association was unclear^55^. Hence, further study on mental health, different levels of diabetes care and BMI is needed to identify the underlying reasons.

Our study has important public health implications. The association of different levels of care along the hypertension cascade and mental distress showed that there are opportunities to improve the provision of mental health treatment. Personalized strategies and interventions can be introduced to target different needs along the cascade. For example, our study pointed out that individuals who were not screened and not diagnosed with hypertension had a higher risk of mental distress. Stigmatization of mental illness and lack of awareness of mental health are important barriers to timely mental healthcare in Malaysia^56^. Negative perception toward mental illness among Malaysians prevents individuals with mental illness from obtaining proper treatment due to fear of discrimination^56^. Therefore, offering hypertension screening in conjunction with mental health screening may help uncover those at risk of mental distress while minimizing stigmatization and discrimination. Financial aids, counselling and regular follow up can be provided for hypertensive patients undergoing treatment to ensure medical adherence and maintenance of normal BP, particularly for the low-income individuals^57,58^. To ensure BP control, there is also a need to raise awareness of the consequences of hypertension and follow up on hypertensive patients who begin their treatment.

A few limitations of this study should be noted. First, using self-reported assessment may lead to measurement bias where the respondents might underestimate or overestimate their mental health status. Second, the causal relationship between hypertension control and mental distress could not be established due to the study's cross-sectional design. The unavailability of data and unable to include of the health status of respondents in the analysis was one of the limitations of this study. Despite the limitations, to our knowledge, this is the first study that examined the relationship between mental distresses with different levels of hypertension care in Malaysia (Fig. S1). Another strength of this study is the moderately large population-based representative sample that consisted of the major ethnic groups in the country. This study's result might apply to neighbouring countries with developing economies.

In conclusion, there is an association between the different levels of hypertension care and mental distress. The incidence of mental illness in Malaysia is rising. Specific mental health interventions could be provided for patients with different needs along the cascade. The application of the hypertension care cascade may help improve mental healthcare provision in the country and other low- and middle-income countries.

## Data availability

Data is available on reasonable request from the corresponding author Prof Dr. Tin Tin Su (tintin.su@monash.edu).

## Supplementary Information

Supplementary Information.
