# Supplementary material for: Mental distress along the cascade of care in managing hypertension
Source: Sci Rep. 2022 Sep 23;12:15910. doi: 10.1038/s41598-022-20020-1 (PMC9508187; doi:10.1038/s41598-022-20020-1)
Supplement: Supplementary file 1 — Supplementary Information. [file 41598_2022_20020_MOESM1_ESM.pdf]

Dimension	Variable	Scale and/or Reference	Items	Default variable name in dataset	‘Questions of self-constructed items’, *answering options*	Recode criteria
**Mental health**	depression<br>anxiety<br>stress	DASS-21[1]	7<br>7<br>7	depression<br>anxiety<br>stress		IF the score obtained is<br>0 – 9 (normal)<br>10 – 20 (mild/moderate)<br>21+ (severe)
**Hypertension care cascade**	screened hypertension	self-constructed items	1	hypertension_1	‘Have you ever had your blood pressure measured by a doctor or other health worker’, *Refused to answer (-8)/Yes (1)/No (2)*	IF<br>hypertension_1=1 (screened)<br>ELSE (unscreened)
	diagnosed hypertension		2	hypertension_2	‘Have you ever been told by a doctor or other health worker that you have raised blood pressure’, *Refused to answer (-8)/Yes (1)/No (2)*	IF<br>hypertension_1=1 AND<br>hypertension_2=1 (diagnosed)<br>ELSE (undiagnosed)
				hypertension_1	‘Have you ever had your blood pressure measured by a doctor or other health worker’, *Refused to answer (-8)/Yes (1)/No (2)*	
	treated hypertension		1	hypertension_4	‘Have you taken any drugs (medication - not Traditional Chinese Medicine (TCM) in the past 2 weeks’, *Refused to answer (-8)/Yes (1)/No (2)*	IF<br>hypertension_4=1 (treated)<br>ELSE (untreated)
	treated among diagnosed patient	self-constructed items	3	hypertension_1	‘Have you ever had your blood pressure measured by a doctor or other health worker’, *Refused to answer (-8)/Yes (1)/No (2)*	IF<br>hypertension_1=1 AND<br>hypertension_2=1 AND<br>hypertension_4=1 (diagnosed & treated)<br>IF<br>hypertension_1=1 AND<br>hypertension_2=1 AND<br>hypertension_4=2 (diagnosed but untreated)
				hypertension_2	‘Have you ever been told by a doctor or other health worker that you have raised blood pressure’, *Refused to answer (-8)/Yes (1)/No (2)*	
				hypertension_4	‘Have you taken any drugs (medication - not Traditional Chinese Medicine (TCM) in the past 2 weeks’, *Refused to answer (-8)/Yes (1)/No (2)*	

**Hypertension care cascade**	controlled hypertension		2	hypertension_4	'Have you taken any drugs (medication - not Traditional Chinese Medicine (TCM) in the past 2 weeks', *Refused to answer (-8)/Yes (1)/No (2)*	IF hypertension_4=1 AND SBP < 140mm.Hg AND DBP< 90 mm/Hg (treated and controlled)
					Reading of systolic blood pressure (SBP) and diastolic blood pressure (DBP)	IF hypertension_4=1 AND SBP > 140mm.Hg OR DBP > 90 mm/Hg (treated and uncontrolled)
**Socio-demographic status**	Age	self-constructed items	1	residents_age	'Your age'	
	Sex			residents_sex	'What is ${Residents_name}'s gender?', *Male (1)/ Female (2)/Other (3)*	
	Ethnicity	self-constructed items	1	residents_ethnicity	'What is ${Residents_name}'s ethnicity?', *Refused to answer (-9)/ Don't know (-8)/ Malay (1)/ Indian (2)/ Chinese (3)/ Bumiputera (4)/ Other (5)/ Orang Asli (8)*	IF residents_ethnicity= 1 OR 4 (Malay)<br>residents_ethnicity= 3 (Chinese)<br>residents_ethnicity=2 (Indian)<br>residents_ethnicity=8 (Aborigine)<br>residents_ethnicity= 5 (others)
	Marital status		1	demographic_11	'What is your marital status', *Refused to answer (-8)/ Never married (1)/ Married (2)/ Separated/Living Apart (Not Divorced) (3)/ Divorced (4)/ Widow(er)(5)/ Cohabiting (6)/ Other (7)*	IF demographic_11=1 (never married)<br>demographic_11=2 (married)<br>demographic _11= 3 OR 4 (separated/divorced)<br>demographic_11= 5 (widow/widower)<br>demographic_11= 6 OR 7 (others)

**Socio-demographic status**	income		3	work_3	‘What is your average personal gross monthly income, in terms of work/salary/pension (RM)’	income = work_3 + work_4 + work_5
				work_4	‘What is your average personal gross monthly income, in terms of money from other’	
				work_5	‘What is your average personal gross monthly income, in terms of money from other sources, e.g., family members outside the household (RM)’	
	education	self-constructed items		education_1	‘What was the highest level of formal schooling completed’, *Never attended school (1)/ Attended but did not finish primary school (2)/ Finished primary school (3)/ started high school (4)/ Finished Form 3 (5)/ Finished Form 5 (6)/ Finished Form 6 (7)/ Started college (Diploma) (8)/ Finished college (Diploma) (9)/ Started university (Degree) (10)/ Finished university (Degree) (11)/ Other (12)/ Do not know (13)/ Refused to answer (14)*	IF education_1 = 1 (no formal education) education_1=2 OR 3 (primary) education_1= 4 OR 5 OR 6 (secondary) education_2= 7 OR 8 OR 9 OR 10 OR 11 (tertiary) education_1= 12 (others)
**Diabetes status**	diabetes status	self-constructed items	2	diabetes_1	‘Have you ever had your blood sugar measured by a doctor or other health worker’, *Refused to answer (-8)/Yes (1)/No (2)*	IF diabetes_1=1 & diabetes_2=1 (yes) ELSE (no)
				diabetes_2	‘Told by a doctor / health worker that you have raised blood sugar or diabetes?’, *Refused to answer (-8)/Yes (1)/No (2)*	

**Body Mass Index**	BMI	self-constructed items	2	hw_5	Height	(hw_8/(hw_5)/100)^2)
				hw_8	Weight	

Table S1. List of dependent and independent variables selected in the analysis.

Reference

1. Lovibond SH, Lovibond PF. Manual for the Depression Anxiety & Stress Scales. 2nd ed. Sydney: Psychology Foundation; 1995.

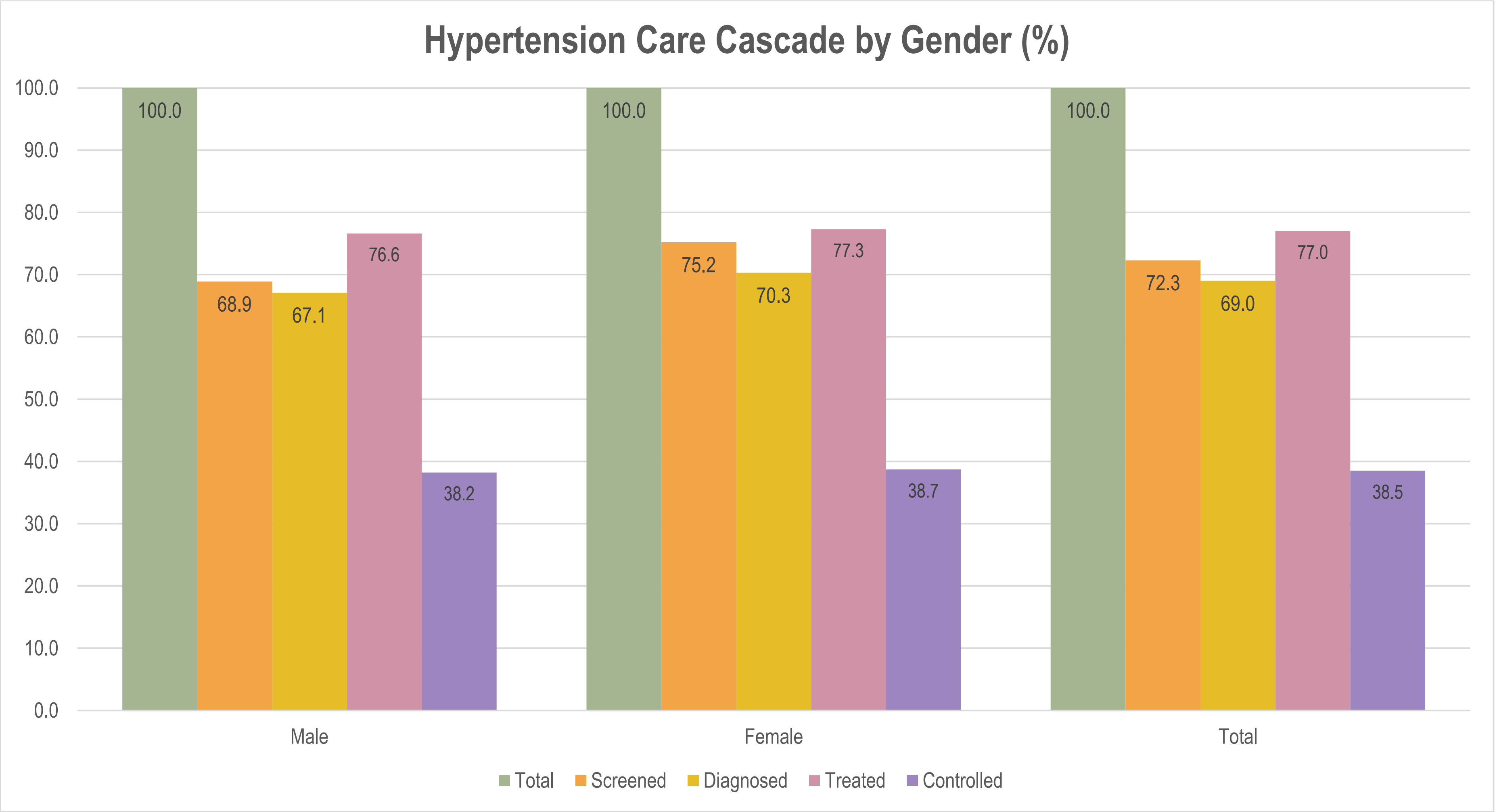

Fig S1. Hypertension care cascade by sex. Distribution of total, screened, diagnosed, treated and controlled hypertension care continuum by gender in percentage (denominator: previous care continuum).

Variables	Male (N=2,915)		Female (N=3,616)		Total (N=6,531)		χ^2, p-value
	n (%)	95% CI (%)	n (%)	95% CI (%)	n (%)	95% CI (%)	
**Screened hypertension**							
Screened	2,007 (68.9)***	(67.2, 70.6)	2,718 (75.2)***	(73.8, 76.6)	4,725 (72.3)	(71.2, 73.4)	32.173, p<0.001
Not screened	908 (31.1)***	(29.4, 32.8)	898 (24.8)***	(23.4, 26.2)	1,806 (27.7)	(26.6, 28.8)	
**Diagnosed hypertension**							
Diagnosed	1,347 (46.2)***	(44.4, 48.0)	1,912 (52.9)***	(51.3, 54.5)	3,259 (49.9)	(48.7, 51.1)	28.694, p<0.001
Undiagnosed	1,568 (53.8)***	(52.0, 55.6)	1,704 (47.1)***	(45.5, 48.7)	3,272 (50.1)	(48.9, 51.3)	
**Hypertension treatment**							
Treated	1,032 (35.4)***	(33.7, 37.1)	1,478 (40.9)***	(39.3, 42.5)	2,510 (38.4)	(37.2, 39.6)	20.415, p<0.001
Not in treatment	1,883 (64.6)***	(62.9, 66.3)	2,138 (59.1)***	(57.5, 60.7)	4,021 (61.6)	(60.4, 62.8)	
**Treatment status in diagnosed group**							
Treated	1,032 (35.4)	(33.7, 37.1)	1,478 (40.9)	(39.3, 42.5)	2,510 (38.4)	(37.2, 39.6)	0.210, p=0.646
Untreated	315 (10.8)	(10.7, 10.9)	434 (12.0)	(10.9, 13.1)	749 (11.5)	(10.7, 12.3)	
**BP status in treated group**							
Controlled	394 (13.5)	(12.3, 14.7)	572 (15.8)	(14.6, 17.0)	966 (14.8)	(13.9, 15.7)	0.070, p=0.791
Uncontrolled	638 (21.9)	(20.4, 23.4)	906 (25.1)	(23.7, 26.5)	1,544 (23.6)	(22.6, 24.6)	

Table S2. Hypertension care continuum of respondents by gender.

Values are total number (percentage; denominator, N of each column) and 95% confidence interval are presented for male, female and total respondents for different hypertension care continuum; ***: p<0.001; **: p< 0.01; *: p<0.05.

	**Depression**	**Anxiety**	**Stress**
	**AOR (95% CI)**	**AOR (95% CI)**	**AOR (95% CI)**
**Age**			
35 – 49	REF	REF	REF
50 – 59	0.785* (0.620, 0.993)	0.966 (0.783, 1.192)	0.831 (0.602, 1.148)
60 – 69	0.896 (0.697, 1.153)	1.013 (0.808, 1.271)	0.851 (0.594, 1.220)
70 and above	1.005 (0.740, 1.366)	0.997 (0.754, 1.318)	1.159 (0.749, 1.793)
**Sex**			**
Male	REF	REF	REF
Female	1.139 (0.946, 1.371)	1.160 (0.983, 1.368)	1.446** (1.109, 1.886)
**Ethnicity**	*	*	**
Malay	REF	REF	REF
Chinese	1.096 (0.900, 1.336)	0.736** (0.615, 0.881)	1.387* (1.050, 1.832)
Indian	1.588** (1.194, 2.111)	0.970 (0.742, 1.268)	1.883** (1.252, 2.830)
Aborigine	1.784 (0.909, 3.502)	0.839 (0.437, 1.608)	2.426 (0.900, 6.538)
Others	1.395 (0.460, 4.232)	1.029 (0.375, 2.829)	3.524 (0.984, 12.620)
**Marital status**	***	**	***
Never married	REF	REF	REF
Married	0.428*** (0.304, 0.601)	0.575** (0.416, 0.797)	0.330*** (0.217, 0.502)
Others	0.427*** (0.282, 0.646)	0.508** (0.345, 0.748)	0.182*** (0.099, 0.336)
**Education level**	***	***	***
Primary	REF	REF	REF
Secondary	0.798* (0.653, 0.974)	0.768** (0.643, 0.917)	1.213 (0.908, 1.623)
Tertiary	0.482** (0.300, 0.774)	0.559** (0.368, 0.849)	0.418* (0.207, 0.842)
Others	0.252*** (0.152, 0.419)	0.530*** (0.373, 0.755)	0.298** (0.133, 0.663)
**Income group**	***	***	***
Below RM 1,000	REF	REF	REF
RM 1,000-RM1,999	1.337** (1.080, 1.654)	1.562*** (1.301, 1.875)	1.202 (0.842, 1.715)
RM 2,000-RM2,999	2.632*** (2.040, 3.397)	2.271*** (1.797, 2.870)	4.545*** (3.186, 6.486)
RM 3,000 and above	3.494*** (2.668, 4.575)	2.690*** (2.099, 3.449)	6.661*** (4.625, 9.593)
**Diabetes Status**	***	***	***
No	REF	REF	REF
Yes	0.513*** (0.382, 0.691)	0.514*** (0.401, 0.660)	0.311*** (0.177, 0.546)
**BMI**	***	***	***
Underweight/ Normal	REF	REF	REF
Overweight	0.692*** (0.575, 0.835)	0.710*** (0.601, 0.839)	0.399*** (0.301, 0.528)
Obese	0.437*** (0.345, 0.555)	0.485*** (0.395, 0.595)	0.305*** (0.214, 0.436)
**Screened hypertension**	***	***	***
Screened	REF	REF	REF
Not screened	2.027*** (1.692, 2.428)	1.787*** (1.521, 2.099)	1.653*** (1.274, 2.144)
**Constant**	0.362***	0.434***	0.136***
**Observed cases (n)**	4,839	4,867	4,857
**χ^2 (df)**	409.125*** (20)	343.958*** (20)	452.368*** (20)
**-2 log likelihood**	3689.305	4523.075	1959.480
**Cox & Snell R Square**	0.081	0.068	0.089
**Nagelkerke R Square**	0.142	0.108	0.227
**Goodness-of-fit (df)**	33.237*** (8)	16.474* (8)	12.117 (8)

Table S3. The odd ratios of depression, anxiety and stress (outcome variables) in binary logistic regression analysis with socio-demographic, diabetes status and screened (adjusted for age, sex, ethnicity, marital status, education, income, diabetes status and BMI) with the observed cases, χ^2 , -2 log likelihood, R^2 and goodness-of-fit.

Abbreviations: AOR, Adjusted Odd Ratios; BMI: Body Mass Index; CI, Confidence Interval; REF, Reference. Values in parentheses are 95% of confidence interval; ***: p<0.001; **: p< 0.01; *: p<0.05.

	Depression	Anxiety	Stress
	AOR (95% CI)	AOR (95% CI)	AOR (95% CI)
**Age**			
35 – 49	REF	REF	REF
50 – 59	0.743* (0.588, 0.938)	0.912 (0.740, 1.124)	0.762 (0.553, 1.050)
60 – 69	0.868 (0.675, 1.116)	0.967 (0.772, 1.213)	0.792 (0.552, 1.137)
70 and above	0.964 (0.710, 1.310)	0.935 (0.707, 1.237)	1.069 (0.690, 1.657)
**Sex**			**
Male	REF	REF	REF
Female	1.123 (0.934, 1.351)	1.133 (0.962, 1.336)	1.444** (1.108, 1.883)
**Ethnicity**		**	*
Malay	REF	REF	REF
Chinese	1.009 (0.830, 1.227)	0.686*** (0.573, 0.820)	1.273 (0.966, 1.677)
Indian	1.469** (1.109, 1.947)	0.910 (0.697, 1.187)	1.735** (1.156, 2.603)
Aborigine	1.534 (0.782, 3.008)	0.747 (0.389, 1.431)	2.081 (0.773, 5.602)
Others	1.269 (0.421, 3.830)	0.937 (0.343, 2.562)	3.123 (0.870, 11.209)
**Marital status**	***	***	***
Never married	REF	REF	REF
Married	0.399*** (0.285, 0.558)	0.533*** (0.387, 0.734)	0.309*** (0.204, 0.468)
Others	0.407*** (0.270, 0.613)	0.480*** (0.327, 0.704)	0.175*** (0.085, 0.322)
**Education level**	***	***	***
Primary	REF	REF	REF
Secondary	0.840 (0.689, 1.025)	0.814* (0.683, 0.970)	1.303 (0.973, 1.745)
Tertiary	0.474** (0.295, 0.761)	0.564** (0.372, 0.856)	0.411* (0.204, 0.828)
Others	0.232*** (0.140, 0.386)	0.504*** (0.354, 0.717)	0.283** (0.127, 0.631)
**Income group**	***	***	***
Below RM 1,000	REF	REF	REF
RM 1,000-RM1,999	1.381** (1.118, 1.706)	1.609*** (1.341, 1.929)	1.247 (0.875, 1.778)
RM 2,000-RM2,999	2.762*** (2.146, 3.556)	2.358*** (1.869, 2.974)	4.761*** (3.342, 6.784)
RM 3,000 and above	3.651*** (2.796, 4.767)	2.762*** (2.159, 3.533)	7.024*** (4.887, 10.095)
**Diabetes Status**	***	***	***
No	REF	REF	REF
Yes	0.428*** (0.317, 0.577)	0.420*** (0.327, 0.541)	0.244*** (0.138, 0.431)
**BMI**	***	***	***
Underweight/ Normal	REF	REF	REF
Overweight	0.663*** (0.551, 0.797)	0.680*** (0.576, 0.802)	0.372*** (0.281, 0.492)
Obese	0.406*** (0.320, 0.515)	0.440*** (0.367, 0.552)	0.275*** (0.193, 0.393)
**Diagnosed hypertension**			
Diagnosed	REF	REF	REF
Undiagnosed	1.144 (0.954, 1.372)	0.975 (0.831, 1.143)	0.875 (0.672, 1.138)
**Constant**	0.497**	0.629*	0.206***
**Observed cases (n)**	4,839	4,867	4,857
**χ^2 (df)**	353.213*** (20)	294.780*** (20)	439.191*** (20)
**-2 log likelihood**	3645.217	4572.253	1972.657
**Cox & Snell R Square**	0.070	0.059	0.086
**Nagelkerke R Square**	0.123	0.093	0.221
**Goodness-of-fit (df)**	39.321*** (8)	21.614** (8)	17.340* (8)

Table S4. The odd ratios of depression, anxiety and stress (outcome variables) in binary logistic regression analysis with socio-demographic, diabetes status and diagnosed hypertension (adjusted for age, sex, ethnicity, marital status, education, income, diabetes status and BMI) with the observed cases, χ^2 , -2 log likelihood, R^2 and goodness-of-fit.

Abbreviations: AOR, Adjusted Odd Ratios; BMI: Body Mass Index; CI, Confidence Interval; REF, Reference. Values in parentheses are 95% of confidence interval; ***: p<0.001; **: p< 0.01; *: p<0.05.

	Depression	Anxiety	Stress
	AOR (95% CI)	AOR (95% CI)	AOR (95% CI)
**Age**	*		
35 – 49	REF	REF	REF
50 – 59	0.729** (0.578, 0.920)	0.899 (0.730, 1.107)	0.753 (0.547, 1.038)
60 – 69	0.845 (0.657, 1.086)	0.941 (0.750, 1.180)	0.771 (0.537, 1.108)
70 and above	0.930 (0.684, 1.263)	0.898 (0.679, 1.188)	1.039 (0.670, 1.610)
**Sex**			**
Male	REF	REF	REF
Female	1.115 (0.927, 1.341)	1.122 (0.952, 1.323)	1.441** (1.105, 1.879)
**Ethnicity**		**	*
Malay	REF	REF	REF
Chinese	0.925 (0.991, 1.205)	0.670*** (0.560, 0.802)	1.251 (0.949, 1.650)
Indian	1.448* (1.093, 1.919)	0.899 (0.689, 1.174)	1.709* (1.138, 2.567)
Aborigine	1.505 (0.767, 2.952)	0.751 (0.391, 1.440)	2.133 (0.793, 5.740)
Others	1.218 (0.403, 3.681)	0.892 (0.325, 2.445)	2.963 (0.821, 10.697)
**Marital status**	***	***	***
Never married	REF	REF	REF
Married	0.395*** (0.283, 0.553)	0.523*** (0.379, 0.721)	0.305*** (0.201, 0.462)
Others	0.404*** (0.268, 0.609)	0.470*** (0.30, 0.690)	0.172*** (0.084, 0.318)
**Education level**	***	***	***
Primary	REF	REF	REF
Secondary	0.858 (0.703, 1.046)	0.829* (0.695, 0.989)	1.328 (0.991, 1.778)
Tertiary	0.482** (0.300, 0.773)	0.575** (0.379, 0.873)	0.416* (0.206, 0.839)
Others	0.237*** (0.143, 0.393)	0.514*** (0.361, 0.733)	0.289** (0.130, 0.646)
**Income group**	***	***	***
Below RM 1,000	REF	REF	REF
RM 1,000-RM1,999	1.390*** (1.125, 1.717)	1.619*** (1.349, 1.942)	1.254 (0.879, 1.787)
RM 2,000-RM2,999	2.751*** (2.137, 3.543)	2.335*** (1.851, 2.947)	4.717*** (3.310, 6.722)
RM 3,000 and above	3.618*** (2.770, 4.724)	2.722*** (2.127, 3.484)	6.948*** (4.833, 9.990)
**Diabetes Status**	***	***	***
No	REF	REF	REF
Yes	0.397*** (0.295, 0.534)	0.391*** (0.304, 0.502)	0.232*** (0.132, 0.407)
**BMI**	***	***	***
Underweight/ Normal	REF	REF	REF
Overweight	0.652*** (0.542, 0.785)	0.669*** (0.566, 0.789)	0.366*** (0.277, 0.485)
Obese	0.396*** (0.125, 0.502)	0.438*** (0.357, 0.538)	0.269*** (0.188, 0.384)
**Hypertension treatment**		**	*
Treated	REF	REF	REF
Not treated	0.956 (0.795, 1.151)	0.793** (0.675, 0.933)	0.740 (0.565, 0.969)
**Constant**	0.579*	0.764	0.241***
**Observed cases (n)**	4,839	4,867	4,857
**χ^2 (df)**	351.309*** (20)	302.508*** (20)	442.926*** (20)
**-2 log likelihood**	3646.120	4564.525	1968.922
**Cox & Snell R Square**	0.070	0.060	0.087
**Nagelkerke R Square**	0.123	0.095	0.223
**Goodness-of-fit (df)**	44.867*** (8)	24.774** (8)	17.243* (8)

Table S5. The odd ratios of depression, anxiety and stress (outcome variables) in binary logistic regression analysis with socio-demographic, diabetes status and hypertension treatment (adjusted for age, sex, ethnicity, marital status, education, income, diabetes status and BMI) with the observed cases, χ^2 , -2 log likelihood, R^2 and goodness-of-fit.

Abbreviations: AOR, Adjusted Odd Ratios; BMI: Body Mass Index; CI, Confidence Interval; REF, Reference. Values in parentheses are 95% of confidence interval; ***: p<0.001; **: p< 0.01; *: p<0.05.

	Depression	Anxiety	Stress
	AOR (95% CI)	AOR (95% CI)	AOR (95% CI)
**Age**			
35 – 49	REF	REF	REF
50 – 59	0.585* (0.389, 0.880)	0.796 (0.556, 1.140)	0.764 (0.434, 1.345)
60 – 69	0.714 (0.467, 1.091)	0.816 (0.558, 1.191)	0.753 (0.410, 1.384)
70 and above	0.737 (0.452, 1.203)	0.685 (0.441, 1.065)	0.816 (0.400, 1.663)
**Sex**			
Male	REF	REF	REF
Female	0.983 (0.733, 1.319)	1.015 (0.786, 1.309)	1.105 (0.723, 1.688)
**Ethnicity**	***	*	***
Malay	REF	REF	REF
Chinese	1.379* (1.028, 1.849)	0.689** (0.531, 0.896)	1.935** (1.264, 2.962)
Indian	2.500*** (1.697, 3.683)	1.263 (0.883, 1.807)	2.956*** (1.679, 5.204)
Aborigine	2.928* (1.219, 7.031)	0.753 (0.308, 1.845)	3.606* (1.060, 12.262)
Others	1.274 (0.269, 6.031)	0.587 (0.124, 2.778)	4.117 (0.820, 20.667)
**Marital status**	*		*
Never married	REF	REF	REF
Married	0.429** (0.229, 0.803)	0.501* (0.277, 0.907)	0.377* (0.170, 0.836)
Others	0.454* (0.222, 0.927)	0.482* (0.249, 0.933)	0.237** (0.086, 0.650)
**Education level**	***	***	
Primary	REF	REF	REF
Secondary	0.524*** (0.376, 0.731)	0.493*** (0.370, 0.656)	0.648 (0.4-8, 1.030)
Tertiary	0.417* (0.190, 0.917)	0.480* (0.243, 0.945)	0.419 (0.137, 1.282)
Others	0.298** (0.148, 0.599)	0.751 (0.470, 1.202)	0.353 (0.119, 1.048)
**Income group**	***	***	***
Below RM 1,000	REF	REF	REF
RM 1,000-RM1,999	1.442* (1.039, 2.000)	1.806*** (1.372, 2.378)	1.006 (0.580, 1.743)
RM 2,000-RM2,999	2.703*** (1.829, 3.995)	2.858*** (2.015, 4.054)	4.514*** (2.711, 7.516)
RM 3,000 and above	3.715*** (2.410, 5.725)	2.953*** (1.991, 4.378)	4.685*** (2.611, 8.406)
**Diabetes Status**	***	***	***
No	REF	REF	REF
Yes	0.429*** (0.309, 0.595)	0.386*** (0.292, 0.510)	0.254*** (0.140, 0.462)
**BMI**	**	***	**
Underweight/ Normal	REF	REF	REF
Overweight	0.952 (0.707, 1.282)	0.865 (0.669, 1.119)	0.690 (0.454, 1.048)
Obese	0.544** (0.378, 0.784)	0.473*** (0.347, 0.646)	0.432** (0.254, 0.736)
**Diagnosed hypertension treated group**	**	***	*
Treated	REF	REF	REF
Untreated	0.626** (0.441, 0.888)	0.542*** (0.402, 0.732)	0.580* (0.339, 0.991)
**Constant**	0.485	0.849	0.185**
**Observed cases (n)**	2,416	2,434	2,424
χ^2 **(df)**	175.463*** (20)	192.636*** (20)	163.175*** (20)
**-2 log likelihood**	1613.712	2052.490	861.841
**Cox & Snell R Square**	0.070	0.076	0.065
**Nagelkerke R Square**	0.134	0.126	0.189
**Goodness-of-fit (df)**	35.931*** (8)	28.295*** (8)	10.575 (8)

Table S6. The odd ratios of depression, anxiety and stress (outcome variables) in binary logistic regression analysis with socio-demographic, diabetes status and hypertension treatment status in respondents diagnosed (adjusted for age, sex, ethnicity, marital status, education, income, diabetes status and BMI) with the observed cases, χ^2 , -2 log likelihood, R^2 and goodness-of-fit.

Abbreviations: AOR, Adjusted Odd Ratios; BMI: Body Mass Index; CI, Confidence Interval; REF, Reference. Values in parentheses are 95% of confidence interval; ***: p<0.001; **: p< 0.01; *: p<0.05.

	Depression	Anxiety	Stress
	AOR (95% CI)	AOR (95% CI)	AOR (95% CI)
**Age**			
35 – 49	REF	REF	REF
50 – 59	0.617* (0.384, 0.991)	0.844 (0.555, 1.283)	0.751 (0.397, 1.419)
60 – 69	0.808 (0.498, 1.312)	0.972 (0.630, 1.499)	0.751 (0.383, 1.474)
70 and above	0.761 (0.435, 1.330)	0.829 (0.504, 1.363)	0.753 (0.340, 1.667)
**Sex**			
Male	REF	REF	REF
Female	1.101 (0.792, 1.531)	1.054 (0.792, 1.404)	1.186 (0.744, 1.890)
**Ethnicity**	***	**	**
Malay	REF	REF	REF
Chinese	1.372 (0.989, 1.905)	0.701* (0.525, 0.936)	1.653* (1.036, 2.637)
Indian	3.055*** (1.984, 3.703)	1.604* (1.077, 2.389)	3.136*** (1.714, 5.736)
Aborigine	1.927 (0.544, 6.831)	0.440 (0.117, 1.659)	2.432 (0.451, 13.102)
Others	1.472 (0.294, 7.358)	0.704 (0.142, 3.482)	4.040 (0.758, 21.534)
**Marital status**			*
Never married	REF	REF	REF
Married	0.437* (0.204, 0.938)	0.493 (0.241, 1.006)	0.327* (0.131, 0.816)
Others	0.407* (0.173, 0.957)	0.465 (0.212, 1.017)	0.191** (0.061, 0.600)
**Education level**	***	***	*
Primary	REF	REF	REF
Secondary	0.457*** (0.313, 0.666)	0.467*** (0.338, 0.644)	0.528* (0.314, 0.886)
Tertiary	0.379* (0.155, 0.931)	0.561 (0.268, 1.176)	0.317 (0.088, 1.144)
Others	0.241** (0.101, 0.576)	0.765 (0.453, 1.292)	0.231 (0.053, 1.015)
**Income group**	***	***	***
Below RM 1,000	REF	REF	REF
RM 1,000-RM1,999	1.720** (1.189, 2.487)	2.015*** (1.477, 2.748)	1.170 (0.639, 2.144)
RM 2,000-RM2,999	3.100*** (2.017, 4.765)	3.364*** (2.294, 4.932)	4.854*** (2.768, 8.513)
RM 3,000 and above	4.768*** (2.943, 7.725)	3.726*** (2.410, 5.761)	5.928*** (3.126, 11.241)
**Diabetes Status**	***	***	***
No	REF	REF	REF
Yes	0.341*** (0.236, 0.493)	0.283*** (0.206, 0.389)	0.215*** (0.111, 0.414)
**BMI**	**	***	*
Underweight/ Normal	REF	REF	REF
Overweight	1.106 (0.789, 1.548)	1.005 (0752, 1.342)	0.787 (0.496, 1.250)
Obese	0.598* (0.395, 0.904)	0.520*** (0.366, 0.739)	0.460* (0.255, 0.829)
**BP status in treated group**	***	***	*
Controlled	REF	REF	REF
Uncontrolled	2.118*** (1.543, 2.908)	1.932*** (1.479, 2.522)	1.646* (1.066, 2.540)
**Constant**	0.232**	0.432	0.154**
**Observed cases (n)**	1,870	1,884	1,876
χ^2 **(df)**	187.182*** (20)	206.329*** (20)	150.466*** (20)
**-2 log likelihood**	1276.421	1628.771	709.021
**Cox & Snell R Square**	0.095	0.104	0.077
**Nagelkerke R Square**	0.175	0.167	0.210
**Goodness-of-fit (df)**	37.160*** (8)	22.073** (8)	15.383 (8)

Table S7. The odd ratios of depression, anxiety and stress (outcome variables) in binary logistic regression analysis with socio-demographic, diabetes status and blood pressure control status in respondents treated for hypertension (adjusted for age, sex, ethnicity, marital status, education, income, diabetes status and BMI) with the observed cases, χ^2 , -2 log likelihood, R^2 and goodness-of-fit.

Abbreviations: AOR, Adjusted Odd Ratios; BMI: Body Mass Index; BP, Blood Pressure; CI, Confidence Interval; REF, Reference.

Values in parentheses are 95% of confidence interval; ***: p<0.001; **: p< 0.01; *: p<0.05.

	Depression	Anxiety	Stress
	OR (95% CI)	OR (95% CI)	OR (95% CI)
**Screened hypertension**	***	***	***
Screened	REF	REF	REF
Not screened	3.043*** (2.652, 3.491)	2.541*** (2.243, 2.880)	3.350*** (2.770, 4.051)
Constant	0.128***	0.198***	0.049***
χ^2 (df)	245.775*** (1)	208.581*** (1)	152.588*** (1)
-2 log likelihood	5407.647	6473.994	3225.029
Cox & Snell R Square	0.038	0.032	0.023
Nagelkerke R Square	0.064	0.049	0.057
Observed cases (n)	6,426	6,469	6,454
**Diagnosed hypertension**	***	***	***
Diagnosed	REF	REF	REF
Undiagnosed	1.775*** (1.548, 2.035)	1.506*** (1.335, 1.699)	1.882*** (1.549, 2.288)
Constant	0.139***	0.216***	0055***
χ^2 (df)	69.361*** (1)	44.758*** (1)	42.025*** (1)
-2 log likelihood	5584.062	6637.817	3335.592
Cox & Snell R Square	0.011	0.007	0.006
Nagelkerke R Square	0.018	0.011	0.016
Observed cases (n)	6,426	6,469	6,454
**Hypertension treatment**	***	***	***
Treated	REF	REF	REF
Untreated	1.459*** (1.265, 1.683)	1.216** (1.074, 1.377)	1.467*** (1.197, 1.797)
Constant	0.149***	0.238***	0.061***
χ^2 (df)	27.733*** (1)	9.576** (1)	14.224*** (1)
-2 log likelihood	5625.689	6673.000	3363.393
Cox & Snell R Square	0.004	0.001	0.002
Nagelkerke R Square	0.007	0.002	0.005
Observed cases (n)	6,426	6,469	6,454

**Diagnosed hypertension treated group**	*	***	*
Treated	REF	REF	REF
Untreated	0.711* (0.542, 0.932)	0.634*** (0.501, 0.802)	0.565* (0.366, 0.871)
Constant	0.149***	0.238***	0.061***
χ^2 (df)	6.430* (1)	15.502*** (1)	7.539** (1)
-2 log likelihood	2370.421	3010.596	1311.591
Cox & Snell R Square	0.002	0.005	0.002
Nagelkerke R Square	0.004	0.008	0.007
Observed cases (n)	3,203	3,231	3,218
**BP status in treated group**	***	***	
Controlled	REF	REF	REF
Uncontrolled	1.696*** (1.310, 2.197)	1.523*** (1.229, 1.887)	1.425 (0.989, 2.052)
Constant	0.105***	0.181***	0.049***
χ^2 (df)	16.931*** (1)	15.293*** (1)	3.755 (1)
-2 log likelihood	1885.687	2414.386	1088.891
Cox & Snell R Square	0.007	0.006	0.002
Nagelkerke R Square	0.013	0.010	0.004
Observed cases (n)	2,463	2,483	2,472

Table S8. The odd ratios of depression, anxiety and stress (outcome variables) in binary logistic regression analysis with five hypertension care status each (independent variables), unadjusted for age, sex, ethnicity, marital status, education, income, diabetes status and BMI, with the observed cases, χ^2 , -2 log likelihood, and R^2.
Abbreviations: OR, Odd Ratios; BP, Blood Pressure; CI, Confidence Interval; REF, Reference.
Values in parentheses are 95% of confidence interval; ***: p<0.001; **: p< 0.01; *: p<0.05.

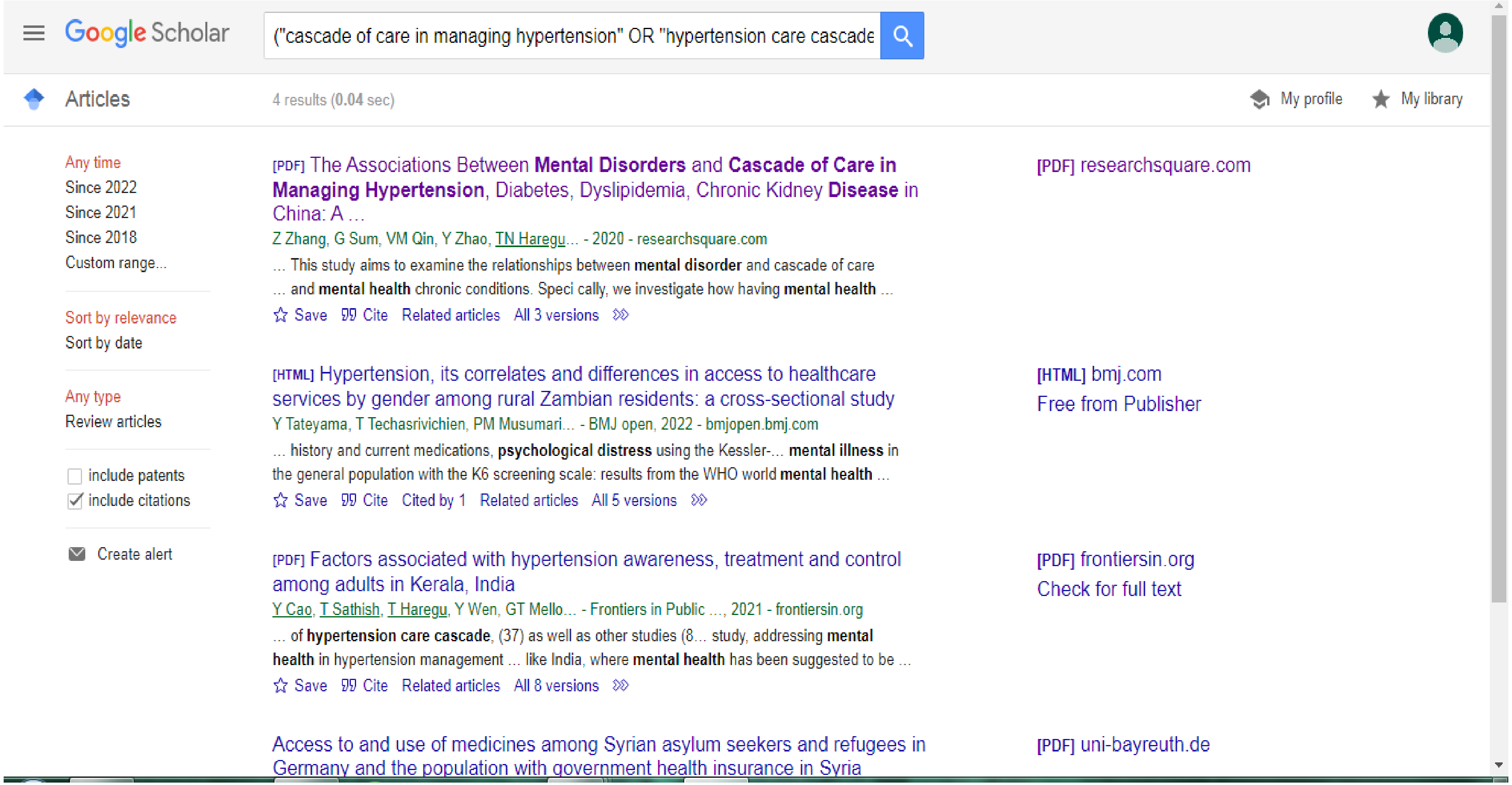

Fig S2. Search result of article on hypertension care cascade and mental disorders. Google scholar link for the search: https://scholar.google.com/scholar?hl=en&as_sdt=0%2C5&q=%28%22cascade+of+care+in+managing+hypertension%22+OR+%22hypertension+care+cascade%22%29+%2B+%28%22mental+health%27+OR+%22mental+illness%22+OR+%22mental+distress%27+OR+%22mental+disorder%22%29&btnG=
